# Physical and nutrient stimuli differentially modulate gut motility patterns, gut transit rate, and transcriptome in an agastric fish, the ballan wrasse

**DOI:** 10.1371/journal.pone.0247076

**Published:** 2021-02-11

**Authors:** Hoang T. M. D. Le, Kai K. Lie, Angela Etayo, Ivar Rønnestad, Øystein Sæle

**Affiliations:** 1 Institute of Marine Research, Bergen, Norway; 2 Department of Biological Sciences (BIO), UiB, Bergen, Norway; Universidade de Vigo, SPAIN

## Abstract

The effects of nutrient and mechanical sensing on gut motility and intestinal metabolism in lower vertebrates remains largely unknown. Here we present the transcriptome response to luminal stimulation by nutrients and an inert bolus on nutrient response pathways and also the response on gut motility in a stomachless fish with a short digestive tract; the ballan wrasse (*Labrus berggylta*). Using an *in vitro* model, we differentiate how signals initiated by physical stretch (cellulose and plastic beads) and nutrients (lipid and protein) modulate the gut evacuation rate, motility patterns and the transcriptome. Intestinal stretch generated by inert cellulose initiated a faster evacuation of digesta out of the anterior intestine compared to digestible protein and lipid. Stretch on the intestine upregulated genes associated with increased muscle activity, whereas nutrients stimulated increased expression of several neuropeptides and receptors which are directly involved in gut motility regulation. Although administration of protein and lipid resulted in similar bulbous evacuation times, differences in intestinal motility, transit between the segments and gene expression between the two were observed. Lipid induced increased frequency of ripples and standing contraction in the middle section of the intestine compared to the protein group. We suggest that this difference in motility was modulated by factors [prepronociceptin (*pnoca*), prodynorphin (*pdyn*) and neuromedin U (*nmu*), opioid neurotransmitters and peptides] that are known to inhibit gastrointestinal motility and were upregulated by protein and not lipid. Our findings show that physical pressure in the intestine initiate contractions propelling the bolus distally, directly towards the exit, whereas the stimuli from nutrients modulates the motility to prolong the residence time of digesta in the digestive tract for optimal digestion.

## 1. Introduction

Ingestion of food initiates a cascade of neural and hormonal signals that prepare the body, and the digestive system in particular, for the tasks ahead and also continues to regulate digestion until the meal has been completely processed [1]. Digestion and absorption of nutrients are mainly facilitated via chemical breakdown by digestive enzymes and aided by mechanical forces through a combination of mixing and transport termed gut motility [1,2]. Gut motility is a complex process that involve coordinated contraction and relaxation of smooth muscle in the gut wall that is closely controlled with enteric neurons and where the typical rhythmic contractile motility patterns are initiated and propagated by special pacemaker cells called the Interstitial cells of Cajal [1,3].

The mixing and propulsion of gut contents along the alimentary tract and the evacuation of unabsorbed and fecal matter can be observed and classified into various motility patterns [4]. Motility patterns fall into two main categories: *i*) non-propulsive contractions, such as segmentation, and *ii*) propulsive contractions, such as peristalsis and migrating motor complexes (MMCs) [2,3]. Each type of contraction plays a specific role, depending on its amplitude, propagation distance and velocity. Segmentations [5–7], also called Standing contractions or stationary contractions, are a set of stationary and rhythmic contractions of the circular muscle, which was first described by Cannon more than 100 years ago [8,9]. This contraction type participates in the digestion (e.g. mixing ingesta with digestive juice) and absorption (i.e. exposing digested gut contents to the absorptive epithelium) [4,9,10]. Propulsive contractions, also referred to as peristalsis or propagating contractions, are a series of annular contractions that produce force to propel luminal contents distally [4,9,11–13]. The propulsive contractions are classified into two main sub-types based on their amplitude and velocity. Ripples are defined as a set of rhythmic and shallow contractions, which propagate for a relatively short distance and at high speed in either the anterograde or retrograde direction [14,15]. It has been suggested that this type of contraction mixes the luminal content for digestion and absorption rather than propelling it [15]. Another sub-type of propulsive contraction is characterized as a series of high-amplitude contractions that propagate for a longer distance than ripples but at a low velocity. In some fish studies these are called slow propulsive contractions [14,16,17], while in studies of humans and other mammals, the accepted term is “high-amplitude propagated contractions” [18,19]. These move luminal contents from one section to the next, thanks to their high amplitude [18,20,21].

Post-prandial segmentation is stimulated and modulated by nutrients present in the gut and the intestinal transit rate is slowed down. In contrast, the presence of inert/non-nutritive ingesta in the intestinal lumen abolishes segmentation and stimulates propulsive contractions; thus, the intestinal evacuation rate increases to egest the indigested content [12,22–26]. Motility patterns differ between feeding and fasting periods. In mammals, when the stomach and intestine are emptied after a meal, the motility shifts to typical myoelectrical patterns that consist of three or four phases, namely the MMCs. This contraction type is made up of a sequence of contractile waves that clean and propel digestive waste, unabsorbed particles and microbiota in the anal direction [13,27–29]. In fish, high frequency of standing contractions with or without ripples, was observed in fed shorthorn sculpin, whereas when the fish was fasting, slow propulsive contractions and retrograde ripples frequently occurred [14].

As in other vertebrates, the gastrointestinal mucosa in fish encompasses a small number of enteroendocrine cells (EEC) and sensory systems. Two components of the sensory system are mechanoreceptors (sensory receptors that respond to mechanical pressure or distortion/stretch) and chemoreceptors (receptors that bind a chemical substance and generate biological signals) [30]. The gastrointestinal hormones/neuropeptides are involved in both local signaling pathways in the enteric nervous system (intrinsic) within the alimentary tract and remote signaling pathways that originate in or communicate with the central nervous system (extrinsic). The enteric nervous system mainly regulates gut motility via afferent, efferent and interneurons that connect with smooth muscle layers of the gut wall and the parasympathetic and sympathetic nervous systems [20,31].

**Fig 1:**
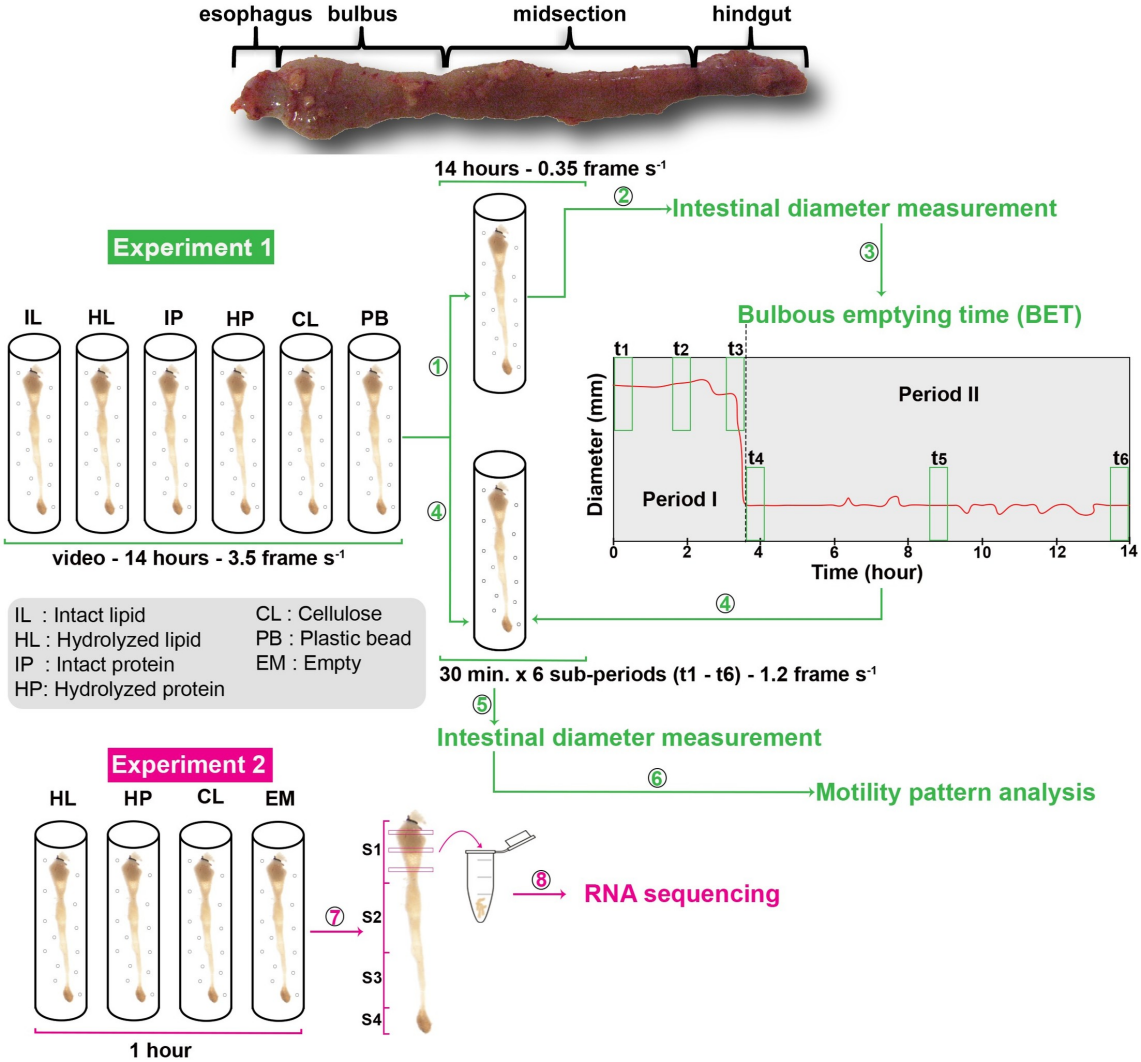
Experimental design and data analysis. The whole intestine and its parts; esophagus, bulbous (Segment 1), mid intestine (Segments 2 and 3) and hind gut (Segment 4). Experiment 1 analyzed the effects of a nutrient or inert bolus on gut motility. Each intestine was administered a bolus into the anterior lumen of one of the five mixtures (IL, HL, IP, HP, and CL) or one plastic bead (PB) before mounting in an individual glass tube containing oxygenated Ringer’s solution. A time-lapse series of images of the intestines was captured at intervals of 3.5 frame s^-1^ for 14.5 h. ①, every tenth frame was subtracted from the original videos (3.5 frames s^-1^) to obtain 0.35 frame s^-1^ videos and ②, intestinal diameters along the intestine on each image frame were measured using NIS-Elements Confocal 4.51.01 software. ③, the data of intestinal diameter was used to calculate bulbous emptying time (BET). Based on BET, times for six sub-periods were defined to select the frames to be in step ④. A standard recovery period of 20 min. was used after mounting the intestines to the glass tube. The period analyzed was from 0 h on the horizontal axis of the Diameter and Time graph, which was set at the 21^st^ min. after the recovery period until 14 h. The sub-period t1 was the first 30 min. after the recovery period; t2 for 30 min. between the 0 h and the BET; t3 for the last 30 min. before the BET; t4, t5 and t6 were the first, middle, and last 30 min., respectively, of the period after the BET until 14 hours after the 0h point. ④, every third frame was subtracted from the original videos for the six sub-periods. ⑤, intestinal diameter was measured on the video frames for each sub-period (t1 –t6) and this data was used to analyze motility patterns in ⑥. Experiment 2 was to collect gut tissue samples for gene expression analysis. Three nutrient bolus types (HL, HP, CL) were selected for this experiment. The isolated intestines were administrated an intraluminal bolus of three selected diets and incubated in Ringer’s solution for 1 h before tissue samples of the bulbous were collected ⑦ and analyzed for RNA sequencing ⑧. S1 –S4, Segments 1–4 of the intestines.

The gut responds to nutrients, both physically (i.e. changes in gut motility induced by luminal presence of food) and at the molecular level (i.e. changes in gut tissue expression of genes related to digestion, absorption and metabolism), as have been well described in mammals [12,22,23,32–36]. The impact of nutrient on control of food intake and energy metabolism has been widely investigated in fish [37–43], and has been reviewed by Conde-Sieira and Soengas [44]. However, it is virtually unknown how the presence of a nutritional or physical (inert/non-nutritional) meal in the gut affect motility and intestinal epithelium metabolism in fish, except for a study on motility patterns in fed and starved sculpin (*Myoxocephalus scorpius*) [14]. The precent study aims to determine how different nutrients regulate both gut motility and gene expression in the intestine. We analyzed changes in motility patterns of isolated whole gut preparations from juvenile ballan wrasse. A bolus of either a nutrient (viscous protein or lipid) or a bolus of an inert suspension containing cellulose or plastic beads were administered *in vitro* to the prepared intestines. Ballan wrasse does not have a stomach, consequently the esophagus ends up in the foregut that has the shape of a bulb. In the buccal cavity the intestine is suspended in a single loop. For the experiments presented here, the intestine was dissected out and the whole organ was used for the study (Fig 1). Differences in gene expression were compared between intestines administered with cellulose versus empty intestines in order to isolate the effect of a mechanical stimulus. Intestines administered with either protein or lipid were compared to cellulose to investigate the effects of chemical stimuli on enterocyte metabolism, excluding the effect of stretch. We also described postprandial motility patterns as responses to administration of the compounds in juvenile ballan wrasse intestines.

## 2. Materials and methods

### 2.1. Treatments

Six treatment mixtures (mix) were prepared, four contained nutrients: intact lipid (IL), hydrolyzed lipid (HL), intact protein (IP), hydrolyzed protein (HP), and two with inert matter: cellulose (CL) and plastic beads (PB). Intact lipid mix was made by stirring a mixture of 80% (by volume) cod liver oil (Møllers Tran, containing omega-3-fatty acids and Vitamin D, see Table 2 in Helland et al., [45] for FFA profile), 15% phosphate-buffered saline (PBS) pH = 8, and 5% Tween 20 (TWEEN^®^ 20, P9416 Sigma Aldrich) to obtain the same viscosity as the hydrolyzed lipid mix. To make 5 mL hydrolyzed lipid mix, we incubated 4 mL cod liver oil with 3.5 mg lipase (Lipase from *Pseudomonas cepacia*—62309 Sigma Aldrich, ≥30 U/mg) dissolved in 750 μL PBS (pH = 8) at 40°C for 5 hours. The pH of the mix was maintained at around 8 by titrating with 5 M NaOH during incubation. The mix was then incubated at 80 °C for 2 hours to deactivate the lipase before mixing with 250 μL Tween. 20. Five mL of intact protein diet was prepared by combining 2 g casein (Casein from bovine milk, C7078, Sigma) with 4 mL 100 mM NH_4_HCO_3_ and 1 mL 1 mM HCl. Hydrolyzed protein mix was prepared by incubating 5 mL intact protein diet with 43.7 mg trypsin (Trypsin Powder, Porcine 1:250, 85450C, Sigma Aldrich) at 37 °C for 20 hours, followed by 80 °C for 2 hours for enzyme deactivation. Five mL of hydrolyzed protein mixture was freshly mixed with 100 μL protease inhibitor [Protease/Phosphatase Inhibitor Cocktail (100X) #5872, Cell Signaling Technology, Inc, The Netherlands] before administration of the bolus into the intestine. Cellulose mix was created using 0.5 mg cellulose (Cellulose microcrystalline powder, 435236 Sigma Aldrich) in 1.5 mL H_2_O, adding a tiny amount of Brillant Blue (Brilliant Blue R, B7920 Sigma-Aldrich) as a color maker. These five mixes were made and stored at– 20 °C for use within a week. The premade mixes were thawed and warmed up at 14 °C before being administered as a single bolus into the intestines. The plastic beads were rinsed in ddH_2_O before being administered.

### 2.2. Animals and tissue preparation

Ballan wrasse juveniles were supplied by a commercial fish farm (Marine Harvest Labrus, Øygarden, near Bergen, Norway) (see Animal and Tissue Preparation in [17]). Fish weighing 20–30 g were transferred from the Marine Harvest farm to the Institute of Marine Research (Bergen, Norway) laboratory and were fasted for one day prior to the experiments.

On the day of the trial, the fish were anesthetized in 0.05 mg/mL neutralized tricaine methanesulfonate (MS222) dissolved in sea water prior to euthanized spinal cord lesion using a scalpel and removal of the intestine. The eviscerated intestine included esophagus and anus with the surrounding skin, leaving the whole intestine intact. Eviscerated intestines (5–9 cm length) were immediately immersed in Ringer’s solution (pH = 7) according to Le *et al*. [17]. The luminal content was gently flushed out with Ringer’s solution. The intestines were then administered a single bolus of one of the five mixes (IL, HL, IP, HP, and CL as described in section *Treatments*) or one plastic bead (2–3 mm diameter and 20–30 mg weight) to mimic ingestion of a meal of 0.1% body weight [46]. The prepared intestines were rapidly mounted in individual glass tubes containing 25 mL oxygenated Ringer’s solution at 14 °C. Thereafter, the intestines were carefully stretched out longitudinally inside the tube with the oral opening closed and the anus open according to Le *et al*. [17] and incubated for 14h for experiment one and 1h for experiment 2 (Fig 1) below.

### 2.3. Experiment 1: Description of motility patterns

#### 2.3.1. Image acquisition

Based on our observations, regular motility was present in the intestines within 10–20 min. after they had been mounted in the medium. Thus, the activity of the intestines was recorded for a total of 14 hours after a 20-min.-acclimatation period to examine the effects of the different nutrients on intestinal motility. The 14-hour duration was chosen for Experiment 1 according to the reported *in vivo* passage rate of ballan wrasse juveniles, in which ingested feed took 10–14 h to pass through the alimentary tract [46]. A time-lapse series of images of intestines was captured during the experiment using a camera (Nikon DS-Fi3) with a macro lens (Nikon, AF Micro-Nikkor 60mm f/2.8D), at a resolution of 1024×768 pixels. The capture of the time-lapse series was controlled with the NIS-Elements Confocal 4.51.01 software and captured 3.5 frames s^-1^. In this experiment, we used six treatments (IL, HL, IP, HP, CL, and PB) with six replicates, and six intestines were processed in parallel in each video. The videos were then used to determine the time at which the administered bolus was transferred from the bulbous—Segment 1 to the downstream intestinal sections and also to examine the intestinal motility patterns (Experiment 1, Fig 1)

#### 2.3.2 Time of emptying the bulbous (Segment 1)

The bulbous is a morphologically distinct feature of the anterior gut of the ballan wrasse, with an expanded diameter relative to more posterior regions (Fig 1). Here we defined the time of emptying the bulbous (bulbous emptying time—BET) as the time at which Segment 1 propelled the bolus to the next section and reduced its average diameter to its minimum value. First, we measured the diameters along the intestines during 14 h of each trial. The method used to quantify changes in width along the whole length of the intestines over time has been fully described in Le *et al*. [17]. Briefly, every tenth frame was extracted from the original videos (3.5 frames s^-1^) to obtain 0.35 frame s^-1^ videos (① in Fig 1) and calibrated for length and time before being analyzed using the NIS-Elements Confocal 4.51.01 software. A threshold for intensity was manually selected to cover the whole intestinal area on each frame. Background noise, generated by air bubbles and equipment accessories, was removed using the “restrictions” functions in the Nis-Element software, whereby the size of the intestine is defined on the basis of pixel recognition and the program removes items in images outside of the intestine. The diameter of the intestine was measured at every one pixel using the function “Automated measurement” in the NIS-Elements software and a diameter-matrix was produced. The diameter-matrix, which is a numerical array of gut diameters along the intestine for 14 hours was used to generate spatio-temporal (ST) maps and to calculate the average width of Segment 1. In this study, we defined the four ballan wrasse intestine segments as the average ratios of segment length/total intestine length for Segment 1 (bulbous), Segment 2, Segment 3, and Segment 4 (hindgut), i.e. 0.39, 0.23, 0.23, and 0.15, respectively, according to the morphological description by Le *et al*. [46] (on Fig 1) and Le *et al*. [17]. The average width of Segment 1 (AW1) in each frame in the 14-hour video was calculated as the mean value of diameters at the pixels covering a length of 39% of the anterior intestine. The 5^th^ percentile value of the AW1 for 14 hours (α) was defined using “quantile” function in R [quantile (data of AW1 in 14 h, 0.05)]. The empty bulbous was assumed to represent an average width which was equal to or smaller than α and named as “bulbous emptying width—BEW”. The BEW values occurred at various points in time during the 14-hour period. Thus, the bulbous emptying time (BET) was defined as the time when the first BEW value occurred after starting to record the treatments.

BET was first examined in three fish using intestinal diameters which were measured at five interval values of 3.5, 1.2, 0.7, 0.35, and 0.02 frames s^-1^ for 14 hours. The results for BET evaluated in the videos at equal or more than 0.35 frames s^-1^ did not differ. The ST maps constructed from videos at 0.35 frames s^-1^ represented well the pattern of change in intestinal width. Thus, we measured intestinal diameter in 14-hour videos at 0.35 frames s^-1^ in order to examine BET. All BETs were verified on the time-lapse videos and the ST maps.

#### 2.3.3. Analysis of motility patterns

Gut motility patterns were analyzed for two defined periods: period I was from 0 h (i.e. 20 min. after the insertion of a bolus) to BET, Bulbus emptying time, and period II was from BET to 14h post-starting point. Motility patterns in each period were analyzed from three 30 min. sub-periods based on the experimental design in [14,47]. The first sub-period (t1, Fig 1) was the first 30 min. of the recording. The second was the 30 min. halfway between 0 h and the BET (t2, Fig 1). The third covered the last 30 min. before the BET (t3, Fig 1). Contractions defined within the three sub-periods t1 –t3 were used to analyze motility patterns (frequency, amplitude, duration, propagation direction, distance, and velocity) for period I, when ingesta remained at the bulbous (Segment 1). The fourth (t4), fifth (t5) and sixth (t6) sub-periods (Fig 1) were the first, middle, and last 30 min., respectively, of the period after the BET until 14 hours after the 0-h point. For the intestines that had a BET less than or equal to 1.5 hours, all frames within 0 h and BET were selected for the analysis of motility patterns.

Video frames for each intestine were extracted for six (or four for a BET of less than 1.5 hours) sub-periods and at intervals of 1.2 frames s^-1^ (according to the test by Le *et al*. [17]). Intestinal diameters were measured along the intestines (as mentioned in the section *Time of emptying the bulbous (Segment 1)*, above) for analysis of motility patterns. The classification builds on our previous study where we characterized the motility patterns of ballan wrasse intestines into three types of contractions; standing contractions, ripples, and slow propulsive contractions [17]. Standing contractions are contractions propagating a distance equal to or < 1.0 mm. Contractions with a propagating distance longer than 1.0 mm were evaluated as ripples or slow propagating contractions. To differ between ripples and slow propagating contractions a linear correlation model was applied to find the regression between intestinal position (response vector) and time (a series of terms which specifies a linear predictor for intestinal position). Ripples were defined as contractions which have a coefficient of determination (R^2^) of the linear curve ≥ 0.8, based on the description of D’Antona et al. [15] and Brijs et al. [14]. Slow propagating contractions, which propagate at a slow velocity, were defined with R^2^ < 0.8. Propagating velocity (mm s^−1^) was the absolute value of the slope of the linear curve. Propagating direction for ripples and slow propagating contractions were also identified from the slope, as slope > 0 for anterograde (oral toward anal) direction and slope < 0 for retrograde (anal toward oral) direction.

We defined the types of contractions and their parameters (i.e. frequency, amplitude, propagating direction, distance, duration, and velocity) as described by Le *et al*. [17]. Frequency of contractions (contractions per min at every mm length of intestine—cpm) and propagation direction (the proportion of contractions, propagating in either anterograde or retrograde direction, compared to the total number of contractions) in each period were calculated based on the total number of contractions that occurred within the three sub-periods in each intestinal segment. The remaining parameters (amplitude, distance, duration, and velocity) were presented as a median of the data set of all contractions during the three sub-periods.

#### 2.3.4. Data analysis

There were no differences in either motility patterns or emptying time between IP and HP or HL and IL. Furthermore, the analysis of free fatty acids and amino acids in the feces collected from intestines after 14 hours did not show a significant difference in nutrient composition between intact and hydrolyzed treatments (S1 and S2 Figs). We therefore pooled the data from IP and HP into a group named “protein” and IL and HL into a group “lipid”; and omitted one damaged intestine adminstered HL. BET and motility patterns were thus analyzed for four groups of nutrient boli (protein n = 12, lipid n = 11, cellulose n = 6, and plastic bead n = 6) for two periods (I and II) in four intestinal segments. One-way ANOVA followed by Tukey HSD were used to evaluate the effect of the four bolus treatments on time for BET. A linear mixed models (lme) analysis followed by Tukey HSD were used to compare the frequencies of each contraction type between the four treatment groups and the two defined periods. Amplitude, propagating distance and direction of each contraction type (continuous proportions ranging from 0 to 1) were treated as quasi-binomial response variables and compared, taking treatment or period as predictor variables for each intestinal segment using a generalized linear mixed models (glmmPQL) (R, version 3.4.2 released 2017-09-28, within R studio interphase (version 1.1.383) for Windows. Duration and velocity were treated as exponential variables in glmmPQL models. The lme and glmmPQL models included individual fish IDs as a random factor and the response variables (contraction parameters) as repeated measurements. Changing the contrast where each treatment/period became the intercept of the model was applied to determine the variation in parameter between the four treatments or between the two periods. Kruskal-Wallis test followed by dunnTest (FSA package) was used to compare the parameters (amplitude, duration and velocity) between the three types of contractions. Differences were treated as significant at p < 0.05 for all tests in this study. Amplitude, duration, propagation distance, and velocity are presented as median ± s.e.m., and other data as mean ± s.d.

### 2.4. Experiment 2: Transcriptome sequencing

The analysis of Experiment 1 showed that there was no difference in BET and motility between the intact (IL or IP) and pre-digested (HL or HP) bolus. Nor were there differences in the composition of the free fatty acids or amino acids in the feces collected from intestines during the 14 hours between the intact and hydrolyzed treatments (see S1 and S2 Figs). We therefore selected HL, HP and CL diets to examine the transcriptomic effects of digestible and indigestible bolus content. We also analyzed gene expression in the empty intestines as control. The HL and HP were employed to examine how the gut responds to a nutrient bolus compared to the non-nutritive matter. The indigestible CL was used to evaluate the mechanical effects induced by the physical presence of a bolus on gut activity compared to control and to separate the effects of stretching to that of nutrients in the HL and HP groups. As size of bolus effects peristalsis [48], the intestines administered a bolus of 0.1% body weight, according to Le *et al*. [48] of one of three nutrient bolus types (HL, HP, or CL) and empty intestines were incubated in Ringer’s solution for one hour before tissue samples for transcriptomic analysis were collected. Segment 1 was selected for transcriptomic analysis because it is the main site for digestion [48]. The first segment of the anterior part (about 40% of the total gut length) was cut off, opened by incision and gently washed with Ringer’s solution. The rest of the gut was discarded. Three pieces of tissue (around 20 mg each) were cut transversely from three parts of the first segment and placed in RNAlater^®^ (Sigma-Aldrich, Missouri, USA) for later RNA extraction (Experiment 2, Fig 1).

#### 2.4.1. RNA sequencing

Total RNA was extracted and treated with BioRobot^®^ EZ1 and RNA Tissue Mini Kit (Qiagen, Hilden, Germany) as previously described by Le, Shao (48). RNA quantity and integrity were validated using a NanoDrop ND-1000 UV—vis Spectrophotometer (NanoDrop Technologies, Wilmington, USA) and Agilent 2100 Bioanalyzer and RNA 6000 Nano LabChip kit (Agilent Technologies, Palo Alto, USA) respectively. All samples had 260/230 and 260/280 ratios above 2.0 and 2.2 respectively. The average RNA integrity number (RIN) of all samples was 7.9±0.7. Sequencing and library preparation were performed by the Norwegian Sequencing Centre (www.sequencing.uio.no). DNA libraries were prepared as previously described by [46] using 90 ng total RNA input to the TruSeq Stranded mRNA Library Prep Kit (Illumina, San Diego, California, USA). For multiplexing, standard Illumina adaptors were used. The libraries were sequenced using the NextSeq Illumina platform (Illumina, San Diego, California, USA) according to the manufacturer’s instructions, generating single end 75bp read libraries with an average library size of 25±6 million reads. Raw reads were submitted to the gene expression omnibus https://www.ncbi.nlm.nih.gov/geo/ (accession number GSE129459).

#### 2.4.2. Differential gene expression analysis

Adaptor removing and quality trimming was performed using the TrimGalore 0.4.2 wrapper tool and default parameters. Library quality was investigated using fastQC embedded in the TrimGalore wrapper (https://github.com/FelixKrueger/TrimGalore). Each intestinal RNAseq library was mapped individually to the labrus genome assembly (European Nucleotide Archive accession number: PRJEB13687, http://www.ebi.ac.uk/ena/data/view/PRJEB13687) using the Hisat2 short read aligner version 2.0.4 [49] and the Ensembl gene annotation (Labrus_bergylta.BallGen_V1.95, 11/25/2018, www.ensembl.org). Transcript abundance for the individual libraries was estimated using FeatureCounts [50] of the Subread package (http://subread.sourceforge.net/). Differential expression analysis was performed using the Bioconductor R package (version 3.4.4) DESeq2 (version 1.18.1) [51]. Genes of which fewer than five samples had gene counts below or equal to 10 reads were excluded from further analysis prior to normalization and differential expression analysis. Significantly expressed genes (p < 0.01) were used for further downstream analysis using the DAVID Bioinformatics Resources 6.8 (https://david.ncifcrf.gov/) with default settings (GO, UP_KEYWORDS and KEGG pathway analysis). Heat maps for hierarchical clustering of differentially expressed genes using multi/group comparison were embedded in the Qlucore omics explorer software package version 3.2 (Qlucore AB, Lund, Sweden).

### 2.5 Ethics statement

"Ballan wrasse juveniles were supplied by a commercial fishfarm (Marine HarvestLabrus, Øygarden, outside Bergen,Norway). The fish was reared in accordancewiththe Norwegian Animal Welfare Act of 12 December1974, no. 73, §§22 and 30,amended 19 June 2009. The facility has a general permission to rear alldevelopmentalstages of Labrus berggylta, license numberH ØN0038 provided by the NorwegianDirectorate offisheries (https://www.fiskeridir.no/English)."

## 3. Results

### 3.1. Motility patterns

The wrasse intestines reacted to the presence of a bolus of nutrients via dynamic changes in the intestinal diameter that reflected smooth muscle-driven contractions and relaxations of the gut. Depending on propagation distance and velocity, the contractile activity was classified as either standing contractions, ripples, or slow propulsive contractions. Standing contractions are non-propulsive, while ripples and slow propulsive contractions are two subtypes of propulsive contractions. A Kruskal-Wallis test followed by dunnTest on our data set showed that slow propulsive contractions had higher amplitudes and lasted longer than other contraction types (slow propulsive contractions > ripples > standing contractions, p < 0.0001) (Table 1). Ripples propagated for a shorter distance (p = 0.007) and at a higher velocity than slow propulsive contractions (p < 0.0001).

**Table 1:** Parameters of three types of contractions.

Parameter	Contraction type	Min	Median	Max
Amplitude (%)	Standing contraction	7.7	47.4^a^	77.9
	Ripples	12.7	55.3^b^	79.0
	Slow propulsive contraction	12.4	56.9^c^	81.3
Duration (s)	Standing contraction	1.7	1.8^a^	23.4
	Ripples	1.79	7.2^b^	258.2
	Slow propulsive contraction	1.72	15.3^c^	506.6
Distance (%)	Ripples	1.2	2.2^a^	16.6
	Slow propulsive contraction	1.3	2.5^b^	16.1
Velocity (mm s^-1^)	Ripples	0.04	0.22^a^	0.67
	Slow propulsive contraction	0.001	0.04^b^	0.70

Data was collected in four segments of the intestines from a total of 35 individuals with n = 11 for lipid, n = 12 for protein, n = 6 for cellulose or plastic bead treatment in the Experiment 1. Letters denote significant difference between contraction types for each parameter (Kruskal-Wallis test followed by dunnTest, p < 0.01). The min, median and max values of each parameter and the statistic tests were calculated and analyzed from 606120 standing contractions, 99010 slow propulsive contractions and 11978 ripples. The medians were presented instead of the means because the data had a right skewed distribution.

### 3.2. Effect of nutrients on the time for emptying bulbous (BET)

**Fig 2:**
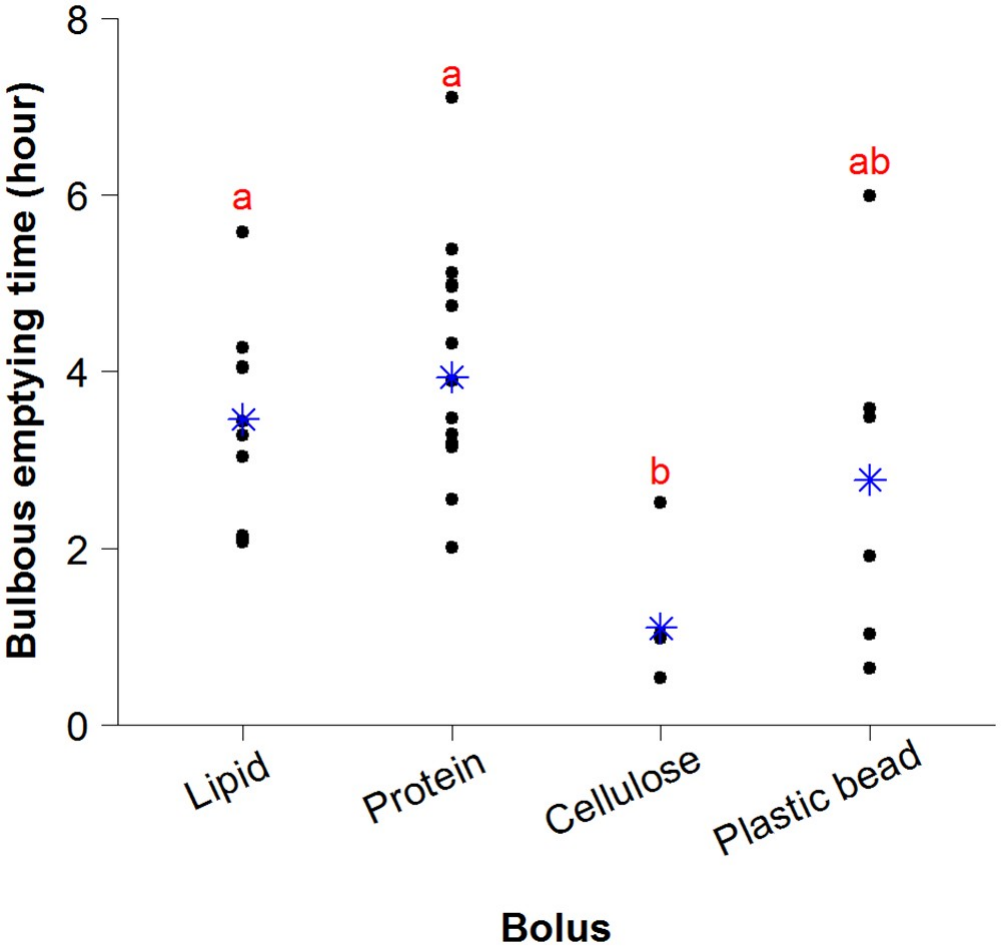
Evacuation time of Segment 1 (the bulbous). Analysis of intestines administered different boli (lipid, protein, cellulose or plastic bead) *in vitro*. Black dots refer to individual intestines, blue stars to mean for each treatment. Letters indicate significant differences in time to emptying of the bulbous between treatments (ANOVA followed by Tukey HSD; n = 11 for lipid, n = 12 for protein, n = 6 for cellulose or plastic bead).

Six types of bolus were inserted into the isolated intestines in order to analyze gut motility. However, since we were unable to completely block endogenous hydrolysis of intact protein and intact lipid, as previously mentioned we pooled data from intact and hydrolyzed lipid treatments into one group for lipid, and data from intact and hydrolyzed protein into one group for protein. The bulbous (Segment 1) emptying time (BET) was 3.4±1.0 h in the lipid treatment and 3.9±1.5 h in the protein group (mean±s.d.) (Fig 2). In contrast, it only took between 0.6 and 2.5 h, with an average of 1.1±0.7 h, for a cellulose bolus to pass through Segment 1. The cellulose group had a faster BET than lipid (ANOVA, p = 0.003) and protein (ANOVA, p < 0.001) (Fig 2). The BET for the plastic bead group was much more variable at between 0.6 and 5.9 hours, and was not different from protein, lipid or cellulose groups.

### 3.3. Effects of bolus composition on motility patterns

#### 3.3.1. Segment 1 (the bulbous)

**Fig 3:**
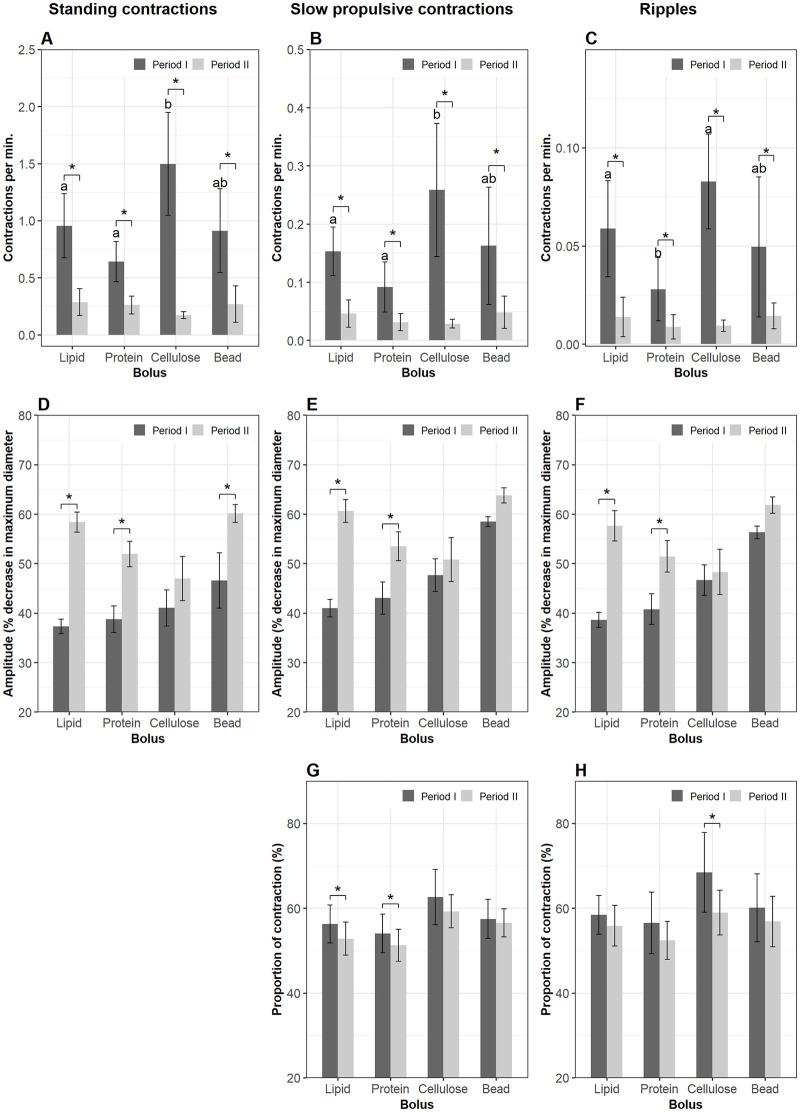
Motility parameters in Segment 1 (the bulbous). Contractions were analyzed in Segment 1 of the intestines when a bolus (lipid, protein, cellulose or plastic bead) was administered into the lumen (period I) and after the bolus left (period II) this segment. Frequency (mean±s.d.) of standing contractions (A), slow propulsive contractions (B) and ripples (C). Amplitude (median±s.e.m.) of standing contractions (D), slow propulsive contractions (E) and ripples (F). Proportion (mean±s.d.) of slow propulsive contractions (G) and ripples (H) which propagate to an anterograde direction. Significant differences (p < 0.05) between the four bolus treatments within period I are annotated by Latin letters and no differences within period II. Asterisks and brackets show a significant difference (p < 0.05) between periods. Data were analyzed using linear mixed-effects models—lme test followed by Tukey HSD in R for (A–C); Generalized Linear Mixed Models via PQL—glmmPQL test for (D–H), with n = 11 for lipid, n = 12 for protein, n = 6 for cellulose or plastic bead.

Bolus composition had significant effects on frequency of contractions on all three motility patterns (Fig 3). When cellulose was present in Segment 1 (period I), it induced more standing contractions (1.5±0.5 contractions per min. for every mm gut length, cpm) compared to lipid (0.9±0.2 cpm) (lme, p = 0.03) and protein (0.64±0.05 cpm) (lme, p = 0.001). The frequency of standing contractions in the plastic bead group was 0.9±0.4 cpm and tended to be lower than that in the cellulose group (lme, p = 0.08). However, the rate of standing contractions in the plastic bead group was not different from that of the lipid and protein treatments (Fig 3A). There was no difference between the lipid and protein group. The presence of cellulose in Segment 1 also induced more propulsive contractions (0.26±0.11 cpm for slow propulsive contractions and 0.08±0.02 cpm for ripples) compared with protein (0.09±0.04 for slow propulsive contractions and 0.03±0.02 cpm for ripples) (lme, p < 0.05) (Fig 3B and 3C). The frequency of slow propulsive contractions in Segment 1 of intestines administered a lipid bolus did not differ from that in the protein group, but it was lower than in the cellulose group (lme, p = 0.04) (Fig 3B). Ripples in the lipid group had a similar frequency to that in the cellulose group but compared to the protein treatment the contraction types occurred at a higher rate (lme, p = 0.04) (Fig 3C). The frequency of propulsive contractions in the plastic bead treatments was not different from the three other groups (Fig 3B and 3C). After ingesta left Segment 1 (period II), the frequency of all three contraction types declined in all four treatments (lme, p < 0.01) (black asterisk, Fig 3A–3C).

The amplitudes of all contraction types in Segment 1 were not different between the four treatments during periods I and II. With a bolus present in Segment 1 (period I) the amplitude increased in all contraction types in both the lipid and protein groups (lme, p < 0.001). However, this was not observed in the cellulose group. The plastic bead induced an increase in amplitude of standing contractions (lme, p = 0.0009) but no change in amplitude of propulsive contractions (Fig 3D–3F).

As long as cellulose was present in Segment 1, 62.6±6.6% of the total slow propulsive contractions and 68.4±9.4% of the total ripples propelled the bolus in an anterograde direction (direction towards the anus). The percentage of anterograde slow propulsive contractions tended to be higher in the cellulose group than in the protein group (glmmPQL, p = 0.06) (Fig 3G). After Segment 1 was empty, the proportion of anterograde slow propulsive contractions in this segment was reduced in the lipid (glmmPQL, p = 0.007) and protein treatment groups (glmmPQL, p = 0.01). However, this parameter did not change in the cellulose or the plastic bead group (Fig 3G). The percentage of anterograde ripples decreased after ingesta left the bulbous in the cellulose treatment (glmmPQL, p = 0.01) but did not change in the three other groups (Fig 3H).

#### 3.3.2. Segment 2

**Fig 4:**
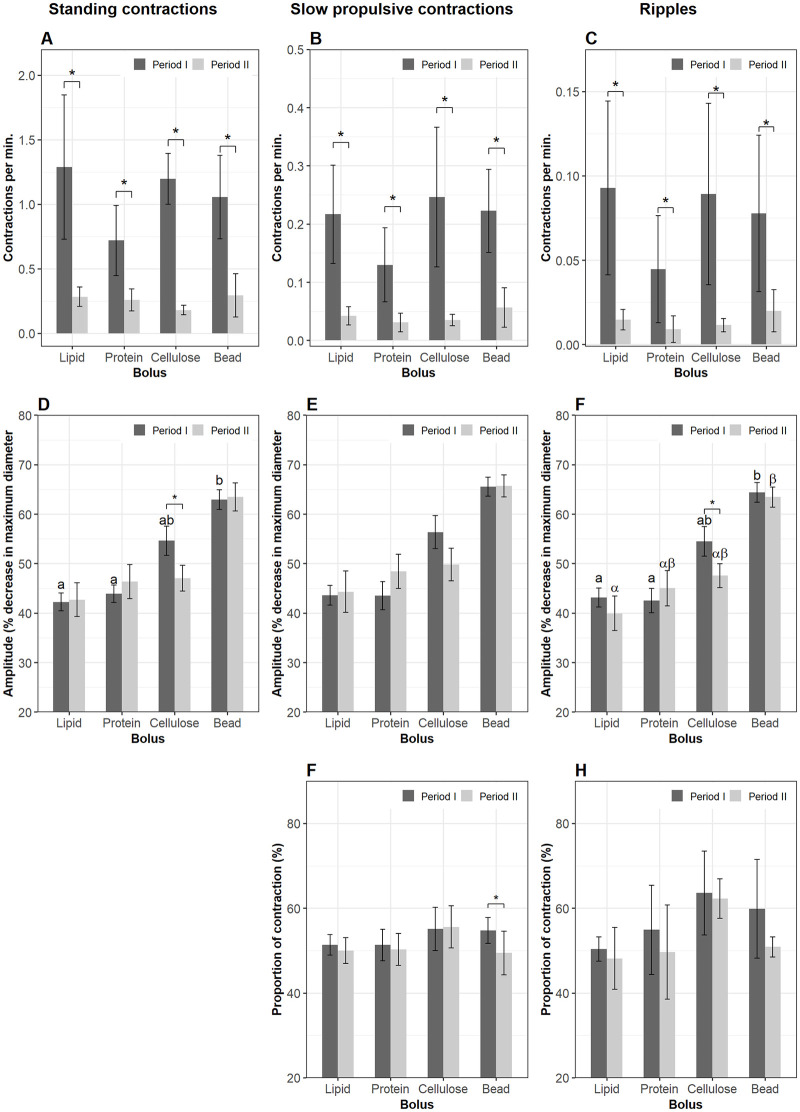
Motility parameters in Segment 2. Contractions were analyzed in Segment 2 of the intestines when a bolus (lipid, protein, cellulose or plastic bead) was administered (period I) and after the bolus evacuated Segment 1 (period II). Frequency (mean±s.d.) of standing contractions (A), slow propulsive contractions (B) and ripples (C). Amplitude (median±s.e.m.) of standing contractions (D) and ripples (E). Proportion (mean±s.d.) of slow propulsive contractions (F) which propagate to an antegrade direction. Significant differences (p < 0.05) between the four bolus treatments within period I are annotated by Latin letters and differences within period II by Greek letters. Asterisks and brackets show a significant difference (p < 0.05) between periods. Data were analyzed using linear mixed-effects models—lme test followed by Tukey HSD in R for (A–C); Generalized Linear Mixed Models via PQL—glmmPQL test for (D–F), with n = 11 for lipid, n = 12 for protein, n = 6 for cellulose or plastic bead.

The frequencies of the three contraction types in Segment 2 were not affected by bolus composition in either period I or II. However, the rate of contractions in Segment 2 was reduced when the boli had left Segment 1 (lme, p < 0.05) (Fig 4A–4C). Standing contractions and ripples had a higher amplitude in the plastic bead treatment compared to the lipid and the protein groups (glmmPQL, p < 0.05) during the residence time in Segment 1. The amplitude of contractions in the cellulose group did not differ from those of the three remaining treatments during either period I or II. During period II the amplitude of ripples in the lipid group was lower than in the plastic bead group (glmmPQL, p = 0.04) (Fig 4E). There was no change in amplitude of contractions in period II compared to that in period I regarding either bolus composition or contraction type. Propulsive contractions in an anterograde direction accounted for over 50% of the total propulsive contractions and the relative proportion of these contractions was not affected by nutrient treatments. The percentage of anterograde slow propulsive contractions in Segment 2 decreased after the plastic bead left Segment 1 (glmmPQL, p = 0.03) (Fig 4C).

#### 3.3.3. Segment 3

**Fig 5:**
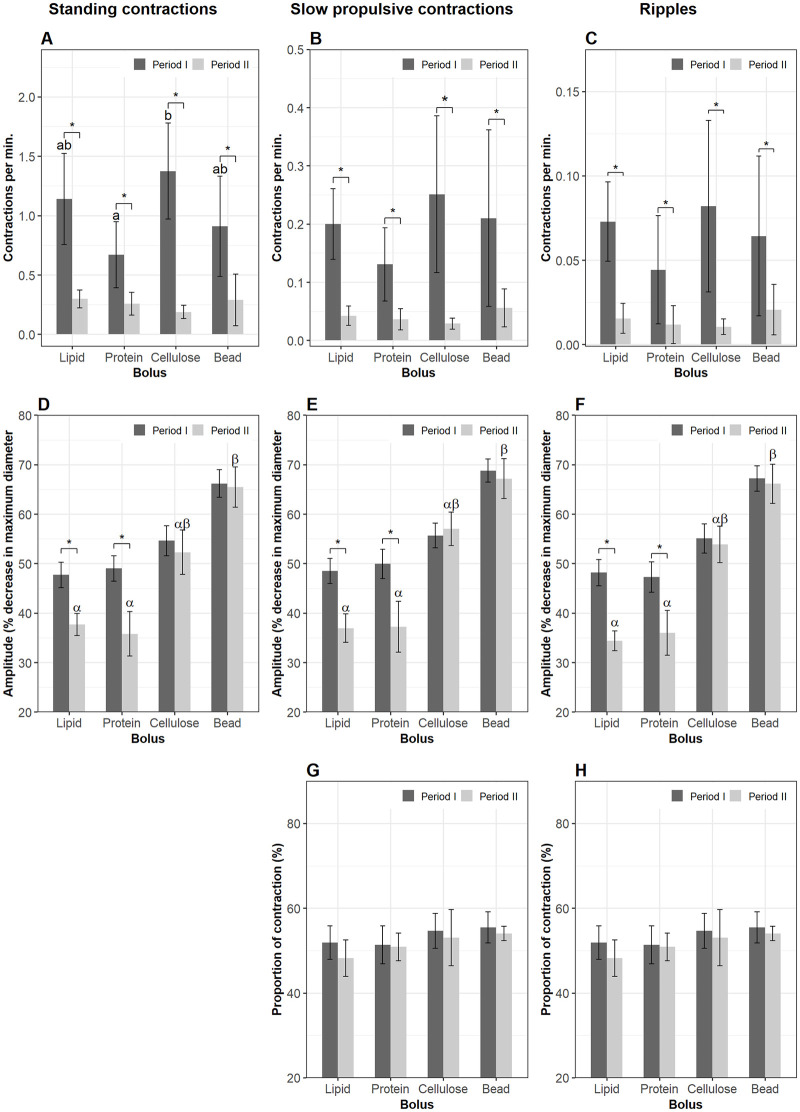
Motility parameters in Segment 3. Contractions were analyzed in Segment 3 of the intestines when a nutrient bolus (lipid, protein, cellulose or plastic bead) presented (period I) and after the bolus left (period II) Segment 1. Frequency (mean±s.d.) of standing contractions (A), slow propulsive contractions (B) and ripples (C). Amplitude (median±s.e.m.) of standing contractions (D), slow propulsive contractions (E) and ripples (F). Significant differences (p < 0.05) between the four bolus treatments within period I are annotated by Latin letters and differences within period II by Greek letters. Asterisks and brackets show a significant difference (p < 0.05) between periods. Data were analyzed using linear mixed-effects models—lme test followed by Tukey HSD in R for (A–C); Generalized Linear Mixed Models via PQL—glmmPQL test for (D–F), with n = 11 for lipid, n = 12 for protein, n = 6 for cellulose or plastic bead.

Cellulose stimulated more standing contractions than protein in Segment 3 during period I (lme, p = 0.04) (Fig 5A). Bolus composition did not affect the frequency of propulsive contraction types (Fig 5B and 5C). The frequencies of the three contraction types diminished after the bolus left Segment 1 in all four treatment groups (Fig 5A–5C). The evacuation of bolus from Segment 1 produced a decrease in amplitude of all contraction types in the lipid and protein groups, and the amplitudes of contractions in these two groups were lower than in the plastic bead group. Amplitude of contraction in the cellulose group did not differ from the remaining groups either before or after ingesta left Segment 1 (Fig 5D–5F). The proportion of anterograde propulsive contractions did not differ between the four bolus groups in either period I or II (data not shown).

#### 3.3.4. Segment 4 (the hindgut)

**Fig 6:**
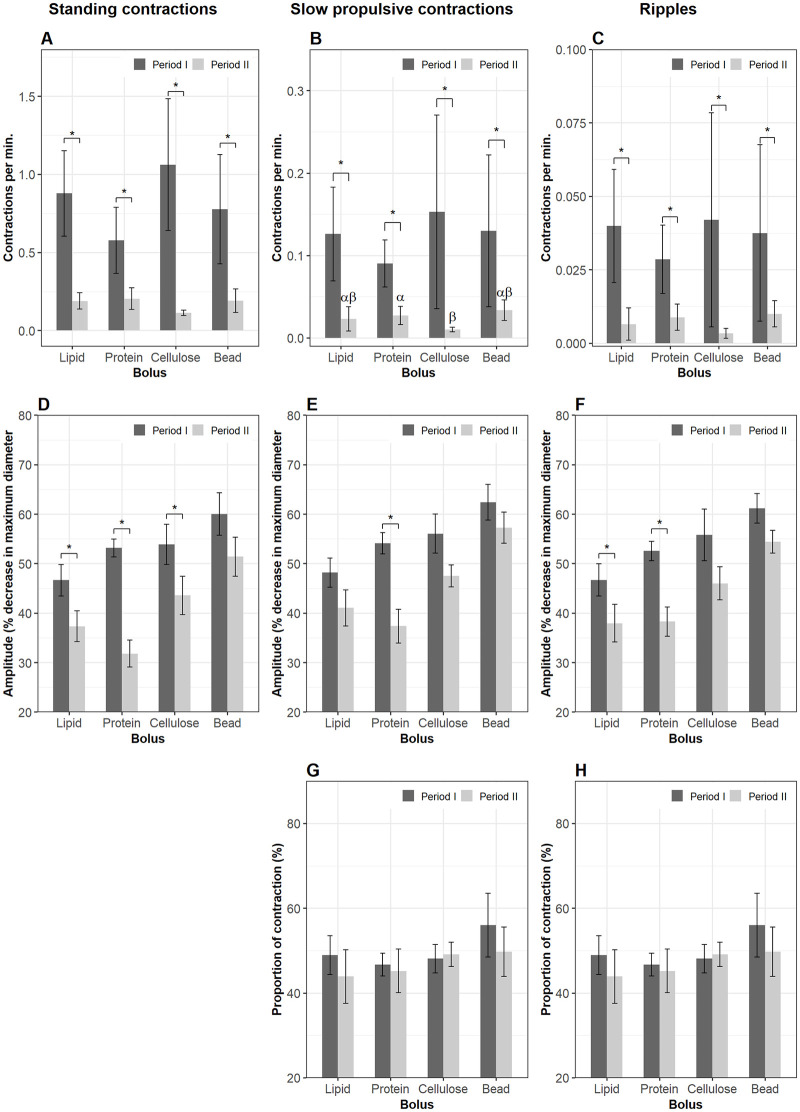
Motility parameters in Segment 4 (the hindgut). Contractions were analyzed in Segment 4 of the intestines when a nutrient bolus (lipid, protein, cellulose or plastic bead) was presented (period I) and after the bolus leaves (period II) Segment 1. Frequency (mean±s.d.) of standing contractions (A), slow propulsive contractions (B) and ripples (C). Amplitude (median±s.e.m.) of standing contractions (D), slow propulsive contractions (E) and ripples (F). Significant differences (p < 0.05) between the four bolus treatments within period I are annotated by Latin letters and differences within period II by Greek letters. Asterisks and brackets show a significant difference (p < 0.05) between periods. Data were analyzed using linear mixed-effects models—lme test followed by Tukey HSD in R for (A–C); Generalized Linear Mixed Models via PQL—glmmPQL test for (D–F), with n = 11 for lipid, n = 12 for protein, n = 6 for cellulose or plastic bead.

As long as the boli was present in Segment 1, there were no effects on the frequency of any of the three contraction types in Segment 4. The evacuation of the boli out of Segment 1 suppressed the frequency of both non-propulsive and propulsive contractions in Segment 4 (Fig 6A–6C) (lme, p < 0.01). The rate of occurrence of slow propulsive contractions in the cellulose group was lower than in the protein in period II (lme, p < 0.05) (Fig 6B). The evacuation of ingesta out Segment 1 reduced the amplitude of standing contractions in three groups, but not in the plastic bead group (Fig 6D); and amplitude of slow propulsive contractions in the protein group (Fig 6E); amplitude of ripples in the lipid and protein groups (Fig 6F).

#### 3.3.5. Comparing motility between the four intestinal segments

The motility characteristics (duration and propagation velocity) were not affected by bolus composition and did not change between periods I and II (data not shown). Variation in frequency of contractions and proportion of anterograde propulsive contractions between the four intestinal segments were affected by bolus composition during period I (before the bolus left Segment 1). Overall, the frequencies of all contraction types in Segment 4 were lower than in the other three segments in all four treatment groups (Table 2).

**Table 2:** Frequency and direction of contractions between the four intestinal segments.

Treatment	Contraction type	Frequency^1^	Anterograde direction^2^
Lipid	Standing contraction	S1 = S4 < S2 = S3	-
	Slow propagating contraction	S1 = S4 < S2 = S3	S1 > S2 = S3 = S4
	Ripple	S1 = S3 < S2 S2 > S3 > S4 S1 = S4	S1 > S2 = S3 = S4
Protein	Standing contraction	S1 = S2 = S3 S1 = S4 S2 = S3 > S4	-
	Slow propagating contraction	S1 = S4 > S2 = S3	S1 = S2 = S3 > S4
	Ripple	S1 = S4 > S2 = S3	S1 > S3 = S4 S1 = S2, S2 = S3 S2 > S4
Cellulose	Standing contraction	S1 > S2 = S4 S1 = S3 > S4 S2 = S3	-
	Slow propagating contraction	S1 = S2 = S3 > S4	S1 > S2 = S3 > S4
	Ripple	S1 = S2 = S3 > S4	S1 = S2 = S3 = S4
Plastic bead	Standing contraction	S1 = S2 = S3 S2 > S4	-
	Slow propagating contraction	S1 = S4 < S2 S1 = S3, S2 = S3	S1 = S2 = S3 = S4
	Ripple	S1 = S4 < S2 S1 = S3, S2 = S3	S1 = S2 = S3 = S4

Variation in frequency of contraction and proportion of contractions propagating in an anterograde direction in the four intestinal segments before ingesta (Lipid, Protein, Cellulose, Plastic bead) left Segment 1 (the bulbous) (period I).

^1^ Frequency of contractions was analyzed using linear mixed models—lme.

^2^ Proportion of contraction propagating in an anterograde direction to the total sum of contractions was analyzed using generalized linear mixed models (glmmPQL).

S1, S2, S3, and S4: Segment 1 (the bulbous), Segments 2, Segment 3 and Segment 4 (the hindgut).

=: No difference (p > 0.05); < or >: Less than or greater than (p ≤ 0.05).

N = 11 for lipid, 12 for protein, 6 for cellulose or plastic bead.

### 3.4. Effects of diets on gene expression

Gene expression in Segment 1 of the intestines fed cellulose was compared to that of empty intestines in order to elucidate the effect of stretch on gut activity. Effects of nutrient and mechanical stimuli on gut metabolism were evaluated by analyzing differential gene expression in Segment 1 of the intestines between protein/lipid and cellulose groups.

#### 3.4.1. Differential gene expression

**Fig 7:**
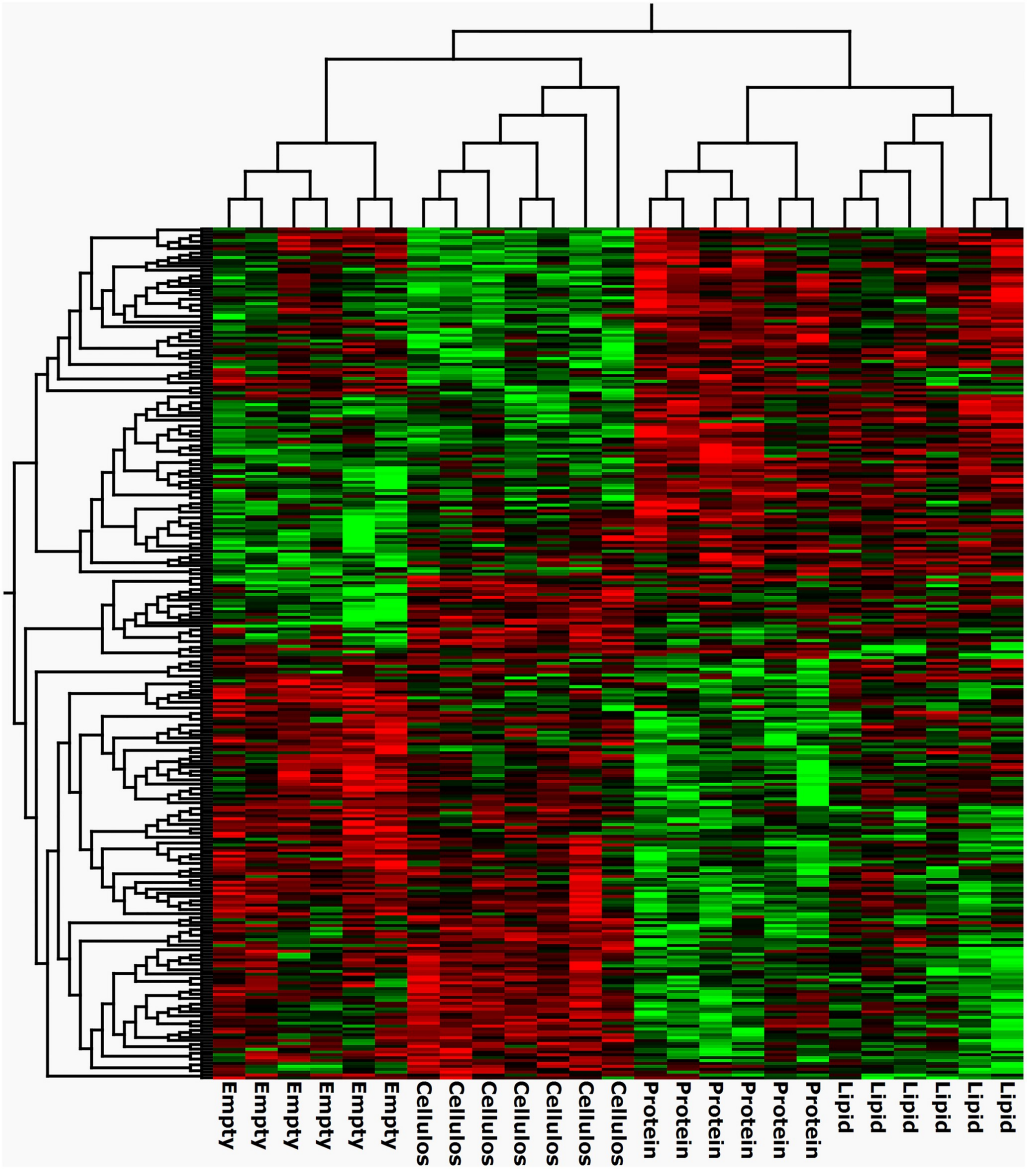
Nutrient and physical stimuli modulates the transcriptome in Segment 1. Hierarchical clustering of differentially expressed (p < 0.01) genes using multi/group comparison embedded in the Qlucore omics explorer. N = 6 for intestines fed with protein or lipid or empty intestines, n = 7 for intestines fed with cellulose.

Genes were filtered using multi/group comparison (p < 0.01), which enables separation of the groups (protein, lipid, cellulose and empty) according to gene expression. Hierarchical clustering revealed two major clusters, one including the two “nutrient” treatments (protein and lipid) groups and one including empty intestines (control) and cellulose-fed (physical stimuli) intestines. A total of 296 genes were clustered for this analysis (Fig 7). There were 178 genes that were differentially expressed (p < 0.01) in intestines with a cellulose bolus compared to empty intestines. A total of 628 and 275 were differentially expressed (p < 0.01) between protein versus cellulose, and lipid versus cellulose respectively (S1 Table). Fewer genes were found to be differentially expressed when protein or lipid were administrated compared to the empty intestines.

**Fig 8:**
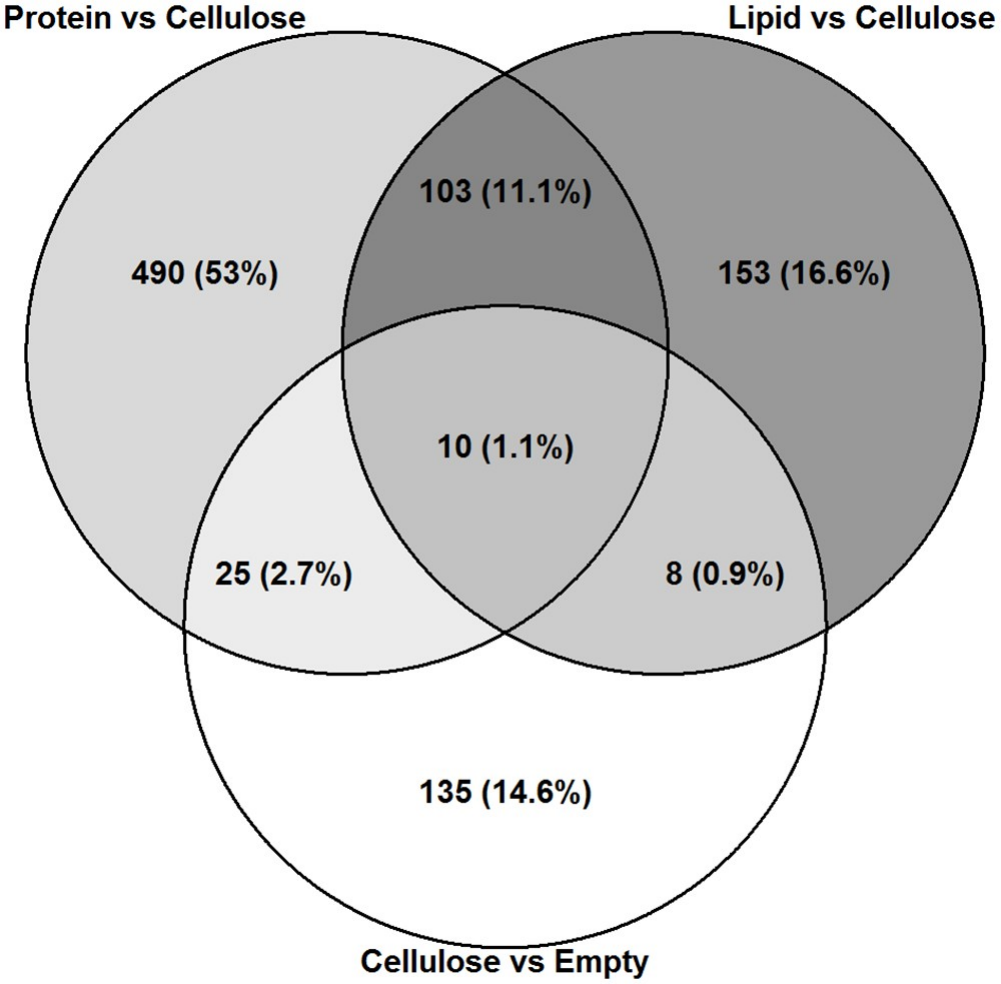
Nutrient stimuli induces changes in gene expression in Segment 1. Venn diagram showing differentially expressed genes (p < 0.01) in intestines administered a protein bolus (n = 6) versus cellulose (n = 7), lipid (n = 6) versus cellulose, and cellulose versus empty groups following DESeq2 analysis of RNA seq data.

In order to identify genes affected by the lipid and protein bolus, we sat the contrast against cellulose in order to remove the effect of stretch. Looking at overlapping genes in the DE gene lists, we found 490 genes affected by protein and 153 by lipid exclusively. We also found 135 genes to be unique for intestines administered cellulose when compared to the empty intestines, while 43 genes were shared with protein and lipid vs cellulose treatments (Fig 8).

#### 3.4.2. Pathway and functional annotation analysis

Pathway analyses were performed using the differentially expressed genes of the different contrasts, including cellulose versus empty, protein versus cellulose, and lipid versus cellulose. Enriched pathways related to calcium were observed in the cellulose versus empty comparison (FDR < 5%) but not in protein versus cellulose and lipid versus cellulose (Table 3).

**Table 3:** Pathways related to muscle contraction and nutrient stimuli were regulated by diet bolus in Segment 1.

Term	Count	%	p value	FDR (%)
**Cellulose versus empty—upregulation**				
Calcium	13	17.1	6.4e-05	0.08
GO:0005509~calcium ion binding	12	15.8	1.4e-04	0.18
**Protein versus cellulose—upregulation**				
Secreted	59	19.5	1.8e-07	2.5e-04
GO:0008528~G-protein coupled peptide receptor activity	5	1.7	2.0e-04	0.29
GO:0007200~phospholipase C-activating G-protein coupled receptor signaling pathway	7	2.3	8.5e-04	1.41
Neuropeptide	6	2.0	3.4e-05	0.05
Opioid peptide	3	1.0	6.4e-04	0.85
GO:0005184~neuropeptide hormone activity	5	1.7	1.54e-03	2.19
GO:0001515~opioid peptide activity	3	1.0	1.67e-03	2.36
GO:0031628~opioid receptor binding	3	1.0	1.67e-03	2.36
GO:0006614~SRP-dependent cotranslational protein targeting to membrane	41	13.5	1.8e-46	2.97e-43
GO:0006364~rRNA processing	53	17.5	1.0e-45	1.70e-42
GO:0006413~translational initiation	44	14.5	4.0e-43	6.69e-40
GO:0019083~viral transcription	41	13.5	9.3e-43	1.56e-39
GO:0000184~nuclear-transcribed mRNA catabolic process, nonsense-mediated decay	41	13.5	1.6e-41	2.72e-38
Ribosomal protein	43	14.2	6.3e-38	8.51e-35
hsa03010:Ribosome	43	14.2	8.0e-38	9.85e-35
Ribonucleoprotein	47	15.5	1.2e-33	1.61e-30
GO:0003735~structural constituent of ribosome	43	14.2	4.5e-32	6.40e-29
GO:0005840~ribosome	38	12.5	8.7e-32	1.13e-28
GO:0022625~cytosolic large ribosomal subunit	28	9.2	4.1e-31	5.33e-28
GO:0006412~translation	44	14.5	7.8e-31	1.31e-27
GO:0044822~poly(A) RNA binding	68	22.4	3.2e-20	4.65e-17
GO:0022627~cytosolic small ribosomal subunit	16	5.3	5.7e-16	7.22e-13
GO:0003723~RNA binding	37	12.2	2.7e-12	3.91e-09
GO:0005925~focal adhesion	23	7.6	4.0e-07	5.19e-04
GO:0005829~cytosol	88	29.0	9.3e-07	1.21e-03
**Lipid versus cellulose—upregulation**				
GO:0006364~rRNA processing	10	9.2	4.8e-06	7.28e-03
GO:0006413~translational initiation	8	7.3	1.6e-05	2.49e-02
GO:0006614~SRP-dependent cotranslational protein targeting to membrane	7	6.4	1.9e-05	2.92e-02
GO:0019083~viral transcription	7	6.4	5.2e-05	0.08
GO:0000184~nuclear-transcribed mRNA catabolic process, nonsense-mediated decay	7	6.4	7.3e-05	0.11
Ribosomal protein	7	6.4	4.4e-04	0.55
hsa03010:Ribosome	7	6.4	4.6e-04	0.55
GO:0022625~cytosolic large ribosomal subunit	5	4.6	5.7e-04	0.67
GO:0006412~translation	8	7.3	7.4e-04	1.12
Ribonucleoprotein	8	7.3	9.6e-04	1.20
GO:0003735~structural constituent of ribosome	7	6.4	1.86e-03	2.32
GO:0005840~ribosome	6	5.5	2.42e-03	2.79

The dashed borders separate clusters of the pathways which relate to the same physiological activity. FDR, False Discovery rate. N = 6 for intestines fed with protein or lipid or empty intestines, n = 7 for intestines fed with cellulose.

The largest number of significantly enriched pathways and functional annotations was observed for protein vs cellulose (FDR < 5%). Both the top enriched pathways (S2 Table) and the largest cluster of pathways affected by protein (S3 Table) were related to the level of ribosomal activity. The neuropeptide signaling pathway, opioid receptor activity pathways, inflammatory response, cytokine activity and G-protein coupled peptide receptor activity were among the many significantly enriched pathways upregulated by protein. Several of the pathways downregulated by protein were related to immune function, such as neutrophil chemotaxis, cellular response to interleukin-1, B cell receptor signaling pathway, and chemokine signaling pathway.

Much fewer significantly enriched pathways were observed in the lipid versus cellulose comparison. Similar to protein, genes involved in pathways related to ribosomal activity and RNA processing were enriched by lipid. Inflammatory response was also found to be significantly enriched by lipid (S3 Table). Comparison of the expression of common genes in intestines between the protein and lipid groups found that inflammatory response, neuropeptide signaling pathway and ribosome were common pathways for the two treatments. The enriched pathways related to muscle contraction and nutrient stimuli are shown in Table 3.

As identified by the pathway analysis, several genes coding for neuropeptide precursors were upregulated following protein and lipid treatment of the intestines (Table 4). None of these genes were differentially affected by cellulose compared to empty intestines. Other relevant neuropeptides such as proopiomelanocortin (*pomc*), neuropeptide Y (*npy*) and peptide YY (*pyy*) were filtered out before differential expression analysis, due to their low transcriptional levels.

**Table 4:** Genes coding for neuropeptides regulated by protein and lipid in Segment 1.

		Protein vs Cellulose		Lipid vs Cellulose	
Gene_ID	Gene_Name	Fold change	Significance	Fold change	Significance
ENSLBEG00000013097	*prepronociceptin (pnoca)*	1.9	*	1.5	-
ENSLBEG00000020351	*neuromedin U (nmu)*	2.5	*	1.3	-
ENSLBEG00000019489	*tachykinin 3 (tac3)*	2.4	**	1.5	-
ENSLBEG00000015930	*prodynorphin (pdyn)*	1.6	*	1.5	-
ENSLBEG00000009804	*neuropeptide B (npb)*	4.0	***	2.2	*
ENSLBEG00000014300	*proenkephalin (penk)*	1.8	**	1.5	*
ENSLBEG00000005406	*cholecystokinin a receptor (cckar)*	2.6	*	2.6	*

* p<0.01,

**p<0.001,

***p<0.0001,

^-^ p ≥ 0.01.

N = 6 for intestines fed with protein or lipid or empty intestines, n = 7 for intestines fed with cellulose.

## 4. Discussion

This study shows that lipid and protein were evacuated from the bulbous (Segment 1) on average of 3.4 to 3.9 h after they were administered into this section of ballan wrasse intestines *in vitro*. We have previously shown that more than 90% of the ingesta left the bulbous 4 h after the feed was administered *in vivo* [46]. This suggest that an *in vitro* approach gives comparable results and provides an estimate of passage rate of ingested matter in the alimentary tract. This points to the autonomous properties of the digestive tract that is maintained even when dissected out of the body, and where many of the reflexes are local and function to optimize function (including motility) towards the digestive processes and where stimuli from the luminal chemical and physical composition are key to maximize the outcome.

The non-nutritive and indigestible cellulose was eliminated from the bulbous (Segment 1) more rapidly than protein and lipid. Slow propulsive contractions seem to play a key role in propelling gut content since these may create a stronger force [18,20,21] and a longer propagation distance than other types of contraction [14]. In the present study, cellulose induced a higher frequency of slow propulsive contractions than lipid and protein, resulting in a faster evacuation of the bolus out of the bulbous. Moreover, over 60% of the cellulose-induced slow propulsive contractions were in an anterograde direction in the bulbous; and this value tended to be higher than that in protein and also tended to be higher than in the lipid group. This suggests that the dominance of anterograde propulsive contractions aids in transferring gut contents in the anal direction; hence, cellulose was propelled out of Segment 1 more rapidly than protein.

Cholecystokinin (CCK), a key hormone in the control of digestion, is released when food is present in the gut in both mammals [52–54] and fish [55–57]. CCK performs its functions related to gastrointestinal motility and digestion mainly through Cholecystokinin A Receptor (CCKAR) in mammals (reviewed by Staljanssens *et al*. [58]) and in fish [59–61]. We have previously found that in ballan wrasse, CCK works mainly through CCKA receptors to suppress propulsive contractions, thus prolonging the residence of food in the bulbous for optimal digestion [17]. In the present study, we found that the presence of either lipid or protein in the lumen upregulated expression of *cckar* (CCKA receptor) which would be activated by CCK to suppress slow propulsive contractions in Segment 1 to delay evacuation of the caloric ingesta from the bulbous. In contrast, there was no change in *cckar* expression in the bulbous of intestines administered cellulose compared to the empty intestines. Instead of activating CCKA receptor to slow the rate of evacuation, cellulose generates a different motility pattern, with increased frequency of propulsive contractions, predominantly anterograde slow propulsive contractions that accelerate the elimination of the indigested content from Segment 1. The upregulation of *cckar* expression in Segment 1 by lipid and protein may be linked to the lower frequency of both ripples and slow propulsive contractions in this segment compared to Segment 2. Conversely, there was no difference in the frequency of these contraction types between the three first segments in intestines fed with cellulose, which showed lower expression of *cckar* than those fed with lipid or protein.

Compared to cellulose, lipid and protein induced an increase in gene expression of *penk* and *npb* which suppress food intake [62–64]. This suggests that the presence of a caloric bolus in the gastrointestinal lumen stimulates enterocytes to secrete and send satiety signals to the central nervous system to inhibit feeding intake [65–67].

Lipid and protein had similar evacuation rates from the bulbous, and both were slower than cellulose. However, it appeared that the response of ballan wrasse intestines to the luminal presence of lipid somewhat differed from that of protein in the bulbous, with regard to both motility patterns and gene expression. Compared to protein, intestines fed lipid generated more ripples, which were suggested to have a function in mixing rather than propelling gut content [15], to facilitate digestion. We have previously shown that protein digestion is more efficient than lipid digestion in the ballan wrasse bulbous (70% and 50% accordingly) *in vivo* [46]. We suggest that the lower digestibility of lipid compared to protein, modulates the intestinal peristalsis to facilitate lipid digestion. Further demonstrated by increased retrograde contractions in Segment 2 in order to propel the partly digested lipid bolus back to Segment 1, the main site of digestion and absorption in the ballan wrasse intestine [46]. Protein digestion is efficient in the bulbous [46], and Segments 2 and 3 will not be stimulated to generate high frequencies of standing contractions for mixing and digesting [68] the already well-digested content received from the previous section. Also, intestines given a protein- bolus apparently will not increase anterograde contractions (i.e. the proportion of anterograde slow propulsive contractions and ripples did not differ between the three first segments) to propel the well-digested protein back towards the bulbous.

Although similar evacuation rates *in vitro*, the *in vivo* difference in digestibility in the bulbous between lipid (50%) and protein (70%) [46] may explain the observed difference in regulation of some pathways between these two groups. Pathways related to regulation of opioid peptide and neuropeptides were enriched in the bulbous by protein but not by lipid. Prepronociceptin (PNOCA) and prodynorphin (PDYN) are prepropeptides which are proteolytically processed to form the various secreted opioid neurotransmitters including dynorphins, enkephalins, endorphins, endomorphins and nociceptin [69,70]. Most of these opiates, such as nociceptin, inhibit gastrointestinal motility [71,72]. The gut-brain peptide neuromedin U (NMU) slows down gastric motility and emptying [73]. The upregulation of *pnoca*, *pdyn* and *nmu* in the bulbous of ballan wrasse intestines fed protein (compared to cellulose) might be related to the lower frequency of all three contraction types in the protein than the cellulose group. Also, the expression of these three genes was altered by protein, but not by lipid (compared to cellulose), which might be related to the observed difference in motility patterns in the two first segments between the lipid and protein groups.

In mammals, the presence of ingesta in the anterior part stimulates activity in the posterior segments of the digestive tract [74,75], and the ingesta is mixed and small amounts of gut content are propelled gradually to the next section until the anterior part is empty [4,76]. Based on this it is likely that the functional role of the high frequency of contractions observed in the wrasse intestinal Segments 2 and 3 within period I was to optimize the digestive process for the small amounts of ingesta which these segments received from the previous section. While the three first segments were mixing ingesta, Segment 4 –the hindgut displayed a “cleaning” activity driven by the slow propulsive contractions to eliminate undigested particles and waste from the previous meals and prepare for the remains of the current meal. When the bulbous was empty (period II), the frequency of all contraction types diminished in Segment 1 due to the absence of ingesta. In gastric vertebrates, the empty stomach will send hunger signals to the brain [13,75,77]. However, it is unknown how hunger is regulated in agastric vertebrates. This puzzle also includes the altricial gastric larval stages of fish that develop stomach only during metamorphosis when they transform from larval to juvenile stages [78]. The frequency of all contraction types was also lower in the midgut (Segments 2 and 3) after Segment 1 was empty. This might be due to absorption of nutrients that did not require a high frequency of contractions and/or an artificial effect when energetic metabolites were gradually diminished after the long incubation time. However, peristaltic activity was observed in intestines incubated for 48 hours.

Genes coding for calcium ion binding proteins were found to be enriched by administering both nutritive or non-nutritive boli. One of these genes, TRPM4 (Transient Receptor Potential Cation Channel Subfamily M Member 4), regulates smooth muscle contractions in a number of organs, including the intestine [79]. This is probably related to the stretching effect induced by the presence of food. While the non-caloric cellulose bolus only induced mechanical transduction, the caloric lipid and protein led to changes in a number of pathways related to cell activity. Most of the gastrointestinal hormones produced by enterocytes and other factors that regulate the digestion and absorption are peptides or proteins [2,3]. Thus, protein and lipid boli upregulated a wide range of pathways related to the activity of ribosomes which, being protein factories [80,81] in enterocytes might be to produce materials for secretion of enzymes, hormones, neurotransmitters and other components that are involved in the processes of digestion and absorption.

The plastic beads, an unbreakable non-nutritive particle, induced either very short or an extended residence time in the bulbous. We hypothesize that the presence of the hard beads in the bulbous signal Segments 2 and 3 to generate high-amplitude standing contractions that serve as a “physiological” brake [4] to delay the propulsion of the indigested meal to the distal sections. Overall, the amplitude of contractions in the plastic bead group was higher than that in other groups and this parameter did not differ between period I and II. This might be an artificial effect where an unbreakable bolus maintains its volume and shape during its entire transit through the intestinal tract. Because of the stability in volume and shape which results in the reliable localization by video-scopy, beads have been widely used in studies of gastrointestinal transit and motility for several decades [82–88]. However, our results show that it is important to consider that the transit time and motility patterns induced by the beads differ significantly from the effect on these factors induced by a nutritious meal. Beads are therefore not a good proxy for intestinal motility during digestion.

## 5. Conclusions

In conclusion, presence of nutrient and physical stimuli in the intestinal lumen regulates both motility patterns and gene expression to maximise digestion and absorption. The non-nutritive cellulose only triggered a stretching effect, increasing propulsive contractions to rapidly move the indigestible bolus from the bulbous towards the hindgut. In contrast, the nutritive protein and lipid reduced the frequency of both non-propulsive and propulsive contractions to prolong the residence of these nutrients in the bulbous, the main site of digestion in ballan wrasse. Protein and lipid upregulate *penk* and *npb*, which are known to initiate satiety signals to the central nervous system to suppress feeding. These nutrients also increased expression of *cckar*, which is known to slow down the evacuation rate of the bulbous. Three other genes; *pnoca*, *pdyn* and *nmu*, were upregulated by protein but not by lipid. This may be related to the differences in signalling pathways and motility patterns observed between the two nutrient groups.

## Supporting information

S1 Fig Protein gel electrophoresis of nutrient bolus and feces in Experiment 1. Lane 1, protein standard with multiple molecular weights (SDS-PAGE Molecular weight standards, Broad Range, Cat.No 161–0317, BIO-RAD); lanes 2, 4, 6, 8, and 10, feces collected from five individual intestines at 14 h after fed a bolus of intact protein; lanes 3, 5, 7, and 9, feces collected from four individual intestines at 14 h after fed a bolus of hydrolyzed protein; lanes 11 and 12, hydrolyzed protein bolus. The samples (nutrient bolus and feces) were diluted 16 times in dH_2_0 before mixing equal amounts of sample with the sample buffer [in volume 9.5:0.5 of Laemmi Sample buffer (BioRad, cat#161–0737) and β-mercapthoethanol (BioRad #161–0710)]. The mixes of samples and buffer sample were heated 5 min, 95 °C. The samples and MW-marker (BioRad#161–0375) were loaded into wells of precast 10% SDS-gels (BioRad #456–1043) in Tris-glycine SDS Running Buffer (#1610772EDU, BioRad) using a BioRad MiniProtean^®^Cell according to the manufacturers instruction. The electrophoresis was run for 30 min. at a voltage of 200 V. The sorted proteins in the SDS-gels were incubated with fixing solution (50% methanol and 10% glacial acetic acid in dH2O) overnight with gentle agitation at room temperature before changing to a staining solution (50% methanol, 10% Glacial acetic Acid, 0.1% Coomassie Brilliant Blue R-250). The staining process was run for 20 min. and followed by destaining in a solution of 40% methanol and 10% glacial acetic acid changing four times for 5, 30, 60, and 10 min. each. Amersham ECL^™^Western Blot Analysis System (# 170–5702620, GE Healthcare) and Chemi Chemiluminiscence Image Capture (Syngene, Cambridge) were used to detect proteins. Signal strength of each specific band was calculated using Gene Tools from Syngene, file version 4.03.10, Synoptics Ltd. The intact protein bolus was made of casein (Casein from bovine milk, C7078, Sigma) which consists of proteins weighting from 19000–23700 Da. The image shows that protein compositions in the feces between intact and hydrolyzed protein were similar and they were similar to the protein compositions of the hydrolyzed protein bolus. The presence of proteins weighting 6500–14400 Da in the feces in the intact protein treatment suggests that the digestion of protein in the intestines was not successful blocked by the protease inhibitor.
(TIF)

S2 Fig Lipid composition in feces collected from the isolated intestines at 14 h after the insert of a bolus in Experiment 1. FFA, free fatty acids; TAG, triacylglycerol. The compositions of lipid were determined using chromatography with the 19:0 methyl ester as an internal standard according to Lie and Lambertsen 1991 [89]. There was no difference in the proportions of free fatty acids in the feces collected from the intestines between the intact lipid (IL) and hydrolyzed lipid (HL) groups [p > 0.05, generalized linear model (GLM)]. The feces in HL group consisted of a lower proportion of triacylglycerol than the feces in IL group (p < 0.05, GLM).
(TIF)

S1 Table Differential gene expression analysis between treatment groups. (XLSX)

S2 Table Functional annotation enrichment analysis using DAVID. (XLSX)

S3 Table Functional annotation clustering using DAVID. (XLSX)
