# Supplementary figures and images for: Physical and nutrient stimuli differentially modulate gut motility patterns, gut transit rate, and transcriptome in an agastric fish, the ballan wrasse
Source: PLoS One. 2021 Feb 11;16(2):e0247076. doi: 10.1371/journal.pone.0247076 (PMC7877642; doi:10.1371/journal.pone.0247076)

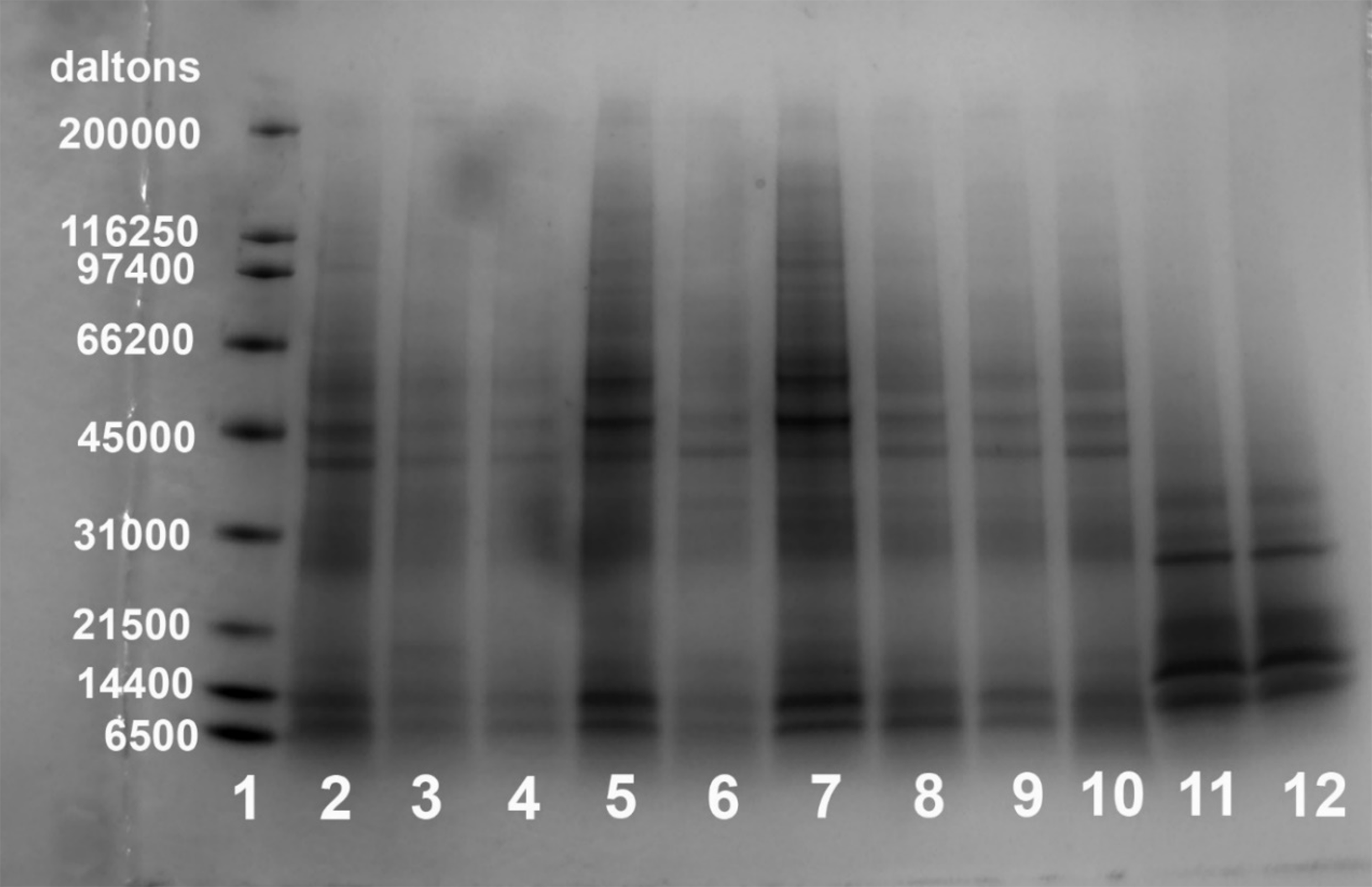

Supplement: S1 Fig — Lane 1, protein standard with multiple molecular weights (SDS-PAGE Molecular weight standards, Broad Range, Cat.No 161–0317, BIO-RAD); lanes 2, 4, 6, 8, and 10, feces collected from five individual intestines at 14 h after fed a bolus of intact protein; lanes 3, 5, 7, and 9, feces collected from four individual intestines at 14 h after fed a bolus of hydrolyzed protein; lanes 11 and 12, hydrolyzed protein bolus. The samples (nutrient bolus and feces) were diluted 16 times in dH20 before mixing equal amounts of sample with the sample buffer [in volume 9.5:0.5 of Laemmi Sample buffer (BioRad, cat#161–0737) and β-mercapthoethanol (BioRad #161–0710)]. The mixes of samples and buffer sample were heated 5 min, 95 °C. The samples and MW-marker (BioRad#161–0375) were loaded into wells of precast 10% SDS-gels (BioRad #456–1043) in Tris-glycine SDS Running Buffer (#1610772EDU, BioRad) using a BioRad MiniProtean®Cell according to the manufacturers instruction. The electrophoresis was run for 30 min. at a voltage of 200 V. The sorted proteins in the SDS-gels were incubated with fixing solution (50% methanol and 10% glacial acetic acid in dH2O) overnight with gentle agitation at room temperature before changing to a staining solution (50% methanol, 10% Glacial acetic Acid, 0.1% Coomassie Brilliant Blue R-250). The staining process was run for 20 min. and followed by destaining in a solution of 40% methanol and 10% glacial acetic acid changing four times for 5, 30, 60, and 10 min. each. Amersham ECL™Western Blot Analysis System (# 170–5702620, GE Healthcare) and Chemi Chemiluminiscence Image Capture (Syngene, Cambridge) were used to detect proteins. Signal strength of each specific band was calculated using Gene Tools from Syngene, file version 4.03.10, Synoptics Ltd. The intact protein bolus was made of casein (Casein from bovine milk, C7078, Sigma) which consists of proteins weighting from 19000–23700 Da. The image shows that protein compositions in the feces between intact and [file pone.0247076.s001.tif]

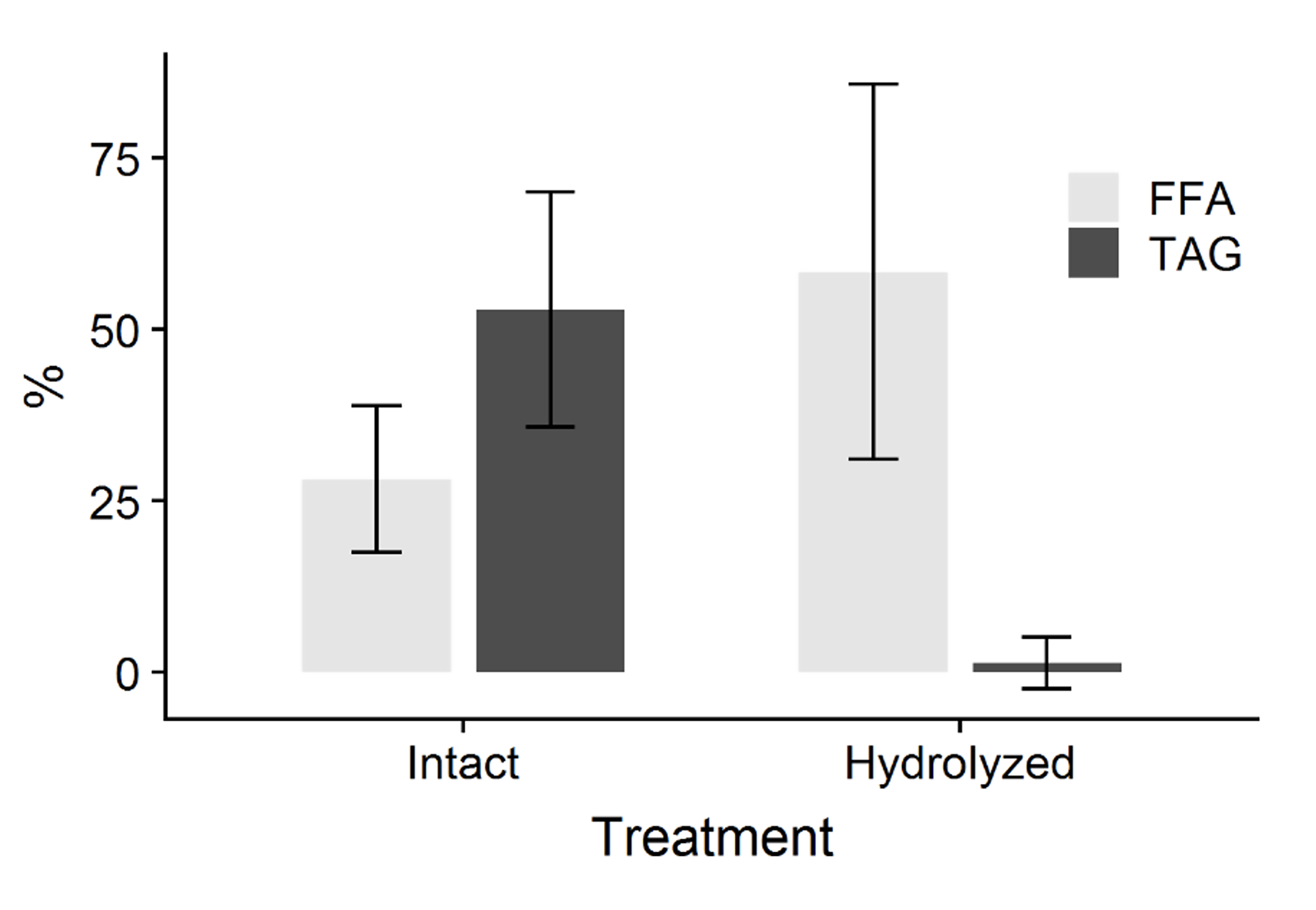

Supplement: S2 Fig — FFA, free fatty acids; TAG, triacylglycerol. The compositions of lipid were determined using chromatography with the 19:0 methyl ester as an internal standard according to Lie and Lambertsen 1991 [89]. There was no difference in the proportions of free fatty acids in the feces collected from the intestines between the intact lipid (IL) and hydrolyzed lipid (HL) groups [p > 0.05, generalized linear model (GLM)]. The feces in HL group consisted of a lower proportion of triacylglycerol than the feces in IL group (p < 0.05, GLM). (TIF) [file pone.0247076.s002.tif]
